# Luzindole and 4P-PDOT block the effect of melatonin on bovine granulosa cell apoptosis and cell cycle depending on its concentration

**DOI:** 10.7717/peerj.10627

**Published:** 2021-03-08

**Authors:** Shujuan Wang, Wenju Liu, Aiyou Wen, Bing Yang, Xunsheng Pang

**Affiliations:** 1 College of Animal Science, Anhui Science and Technology University, Fengyang, China; 2 Anhui Province Key Laboratory of Animal Nutritional Regulation and Health, Fengyang, China; 3 College of Life and Health Science, Anhui Science and Technology University, Fengyang, China

**Keywords:** Melatonin, Melatonin receptor antagonist, Bovine, Granulosa cell, Gene regulation

## Abstract

Granulosa cells play an essential physiological role in mediating the follicle development and survival or apoptosis of granulosa cells dictate the follicle development or atresia. The aim of this study was to investigate the role of high dose (10^−5^ M) and low dose (10^−9^ M) melatonin in bovine granulosa cells, and assess whether MT1 and MT2 inhibiter affect granulosa cells response to melatonin. We found that the high dose (10^−5^ M) and low dose (10^−9^ M) both could act as an essential role in modulating granulosa cells apoptosis, cell cycle and antioxidant. The beneficial effect could be related to that melatonin promoted the expression of *Bcl2*, *Bcl-xl*, *SOD1* and *GPX4*, and inhibited *Bax*, *caspase-3* and *p53* expression. Moreover *P21* expression was decreased in granulosa cells treated with the high dose (10^−5^ M) melatonin and increased in that treated with the low dose (10^−9^ M) melatonin. To further reveal the role of MT1 and MT2 in mediating the effect of melatonin on granulosa cells apoptosis, cell cycle and antioxidant, we found that the luzindole and 4P-PDOT did not affect the effect of high dose (10^−5^ M) melatonin on regulating *Bcl2*, *Bax*, *caspase-3*, *SOD1*, *GPX4* and *p53* expression, while blocked its effect on modulating *Bcl-xl* and *P21*expression. However, luzindole and 4P-PDOT disturbed the effect of low dose (10^−9^ M) melatonin on regulating *Bcl2*, *Bax*, *caspase-3*, *Bcl-xl*, *SOD1*, *GPX4*, and *p53* expression. In conclusion, these results reveal that the effect of low dose (10^−9^ M) melatonin on granulosa cells apoptosis are mediated by MT1 and MT2, and the high dose (10^−5^ M) melatonin affect the granulosa cells apoptosis by other pathway, besides MT1 and MT2. Moreover MT1 and MT2 may work in concert to modulate bovine granulosa cells function by regulating cellular progression and apoptosis.

## Introduction

The mammalian ovary is an important organ for females during their whole reproductive life. It can produce oocytes, giving animals the chance to procreate. However, every oocyte does not have the chance to ovulate, and most of the follicles undergo atresia during their development (Kaipia & Hsueh, 1997). Many factors affect the ovarian follicle development process, such as reactive oxygen species (ROS), disease, endocrine and local autocrine/paracrine systems, as well as the status of granulosa cells (Richards, 2001; Vitt & Hsueh, 2001; Orisaka et al., 2006). Therefore, granulosa cells play an essential physiological role in mediating the oocytes mature and follicle development. Even the survival or apoptosis of granulosa cells dictate the follicle development or atresia (Matsuda et al., 2012; Jiang et al., 2003; Choi et al., 2011).

Granulosa cells act as so important role during folliculogenesis, and many studies focus on exploring the reason of follicular atresia caused by granulosa cells. Granulosa cells can secret estradiol and progesterone which are critical for maintaining ovarian function and inhibiting granulosa cells apoptosis (Arosh et al., 2004; Okamoto et al., 2016). The luteinization of granulosa cells is essential for pregnancy maintenance, which is a temporary organ for secretion of progesterone (He et al., 2016a). During follicles development, granulosa cells completed the preparations for proliferation and differentiation, shifted energy metabolism to glycolysis, and follicles acquired the capacity for estradiol secretion and ovulation (Chen et al., 2020). The granulosa cells within the follicle accompanied by the proliferation, differentiation, and apoptosis during the reproductive period (Chowdhury et al., 2016; Denkova et al., 2014; Chen et al., 2020). Therefore, the apoptosis cause the change of granulosa cells function and disturb the state of follicle development. Meanwhile, the balance between *Bcl-2* family and Bax is crucial for granulosa cells survival and apoptosis. These apoptosis related factors include *Bcl2*, *Bcl-xl*, *Bax*, *caspase-3* and *p53*, which act as crucial roles in regulating granulosa cells apoptosis (Cruz et al., 2014; Fu et al., 2014; He et al., 2016b). Endogenous and exogenous factors producing ROS cause oxidative stress, induce cell membrane and DNA damage, lead to the granulosa cells apoptosis and are involved in antral follicle atresia (Gupta et al., 2006; He et al., 2016d; Kang et al., 2009).

Granulosa cells undergo many negative factors, and how to overcome these disadvantages is very important for granulosa cells to avoid apoptosis. Melatonin is present in follicular fluid, and its concentration is significantly higher compared to that in peripheral blood serum concentration, and it has also been found that there is positive correlation between melatonin concentration and follicular diameter (Yie et al., 1995; Tamura et al., 2009, 2012). The main reason is that melatonin can been secreted by granulosa cells (Tamura et al., 2009; Reiter et al., 2014; Suofu et al., 2017). Recently, melatonin has exhibited the essential role in directly regulating the ovary function. It is well known that melatonin has the benefit of promoting the oocyte maturation, embryos development, as well as hormone secretion of granulosa cells (Dubocovich & Markowska, 2005; Tian et al., 2014; Wang et al., 2012). Moreover, melatonin can mediate the follicle development and protect the integrity of oocytes and granulosa cells and prevent the apoptosis by scavenging the ROS (Tamura et al., 2009; Fatehi et al., 2005; He et al., 2016a; Tanabe et al., 2015). In addition, it is well established that melatonin prevents the apoptosis of granulosa cells by mediating the apoptosis-related genes, including *Bcl2* family, *p53* and *caspase-3*, which are involved in the granulosa cells apoptosis progress (Cruz et al., 2014; Fu et al., 2014; He et al., 2016b; Wang et al., 2012). Although numerous studies have focused the effect of melatonin on granulosa cells, melatonin affecting granulosa cells function through which melatonin receptor is still needed to be investigated.

Melatonin exerts its physiological function primarily through binding to its receptors, MT1 and MT2 (Dubocovich & Markowska, 2005). To confirm the actions of melatonin preventing the apoptosis of granulosa cells and regulating cell cycle are meditated through its receptor, MT1 and MT2. Melatonin receptor antagonist, luzindole and 4P-PDOT were used to further measure the actions of melatonin on granulosa cells, and some crucial genes regulate granulosa cells apoptosis and cell cycle were also determined by qRT-PCR.

## Materials and Methods

### Bovine granulosa cells isolation and culture

Granulosa cells collection was performed as our previously described else where (Wang et al., 2017a, 2017b, 2018; Liu et al., 2018). Bovine ovaries were obtained from Bengbu abattoir (Anhui, China). About 60 bovine ovaries were chose by washing three times using 70% alcohol, and then the ovaries were washed three times by sterile 0.9% NaCl to remove alcohol in this study. The follicular fluid from 3–8 mm antral follicles were chose to isolate the granulosa cells using a syringe and sterile needle puncture method. The follicular fluid also were filtered using a 400 mesh cell strainer. The cell pellets isolated from follicles were digested for 5 min using 0.25% trypsin with 0.025% EDTA (catalog number: 15400-054; Gibco, Grand Island, NY, USA). Then the cell pellets digested were centrifugated at 1,500 rpm for 5 min. The pellets were diluted with Dulbecco’s Modified Eagle Medium (DMEM) (catalog number: 430-2100; Gibco, Grand Island, NY, USA) supplemented with streptomycin (50 µg/ml), penicillin (50 IU/ml) (catalog number: 15070-063; Pen-Strep, Invitrogen, Carlsbad, CA, USA), plasmocin (25 µg/ml, catalog number: ant-pc; Invivogen, San Diego, USA), and 10% fetal bovine serum (FBS, catalog number: SH30070.03; Hyclone, UT, USA). Finally, the separated cells were placed in 60-mm cell culture dishes. The granulosa cells were cultured at 37 °C in an incubator containing 5% CO_2_. In this study, the protocols for the experiment were reviewed and approved by the Institutional Committee on Animal Care and Use at Anhui Science and Technology University, and the experiments were repeated three times independently.

### RNA extraction

To assess the target genes expression level in granulosa cells treated with melatonin within or without 4P-PDOT (catalog number: 134865-74-0; Sigma, Louis, MO, USA) or luzindole (catalog number:117946-91-5; Sigma, Louis, MO, USA), total RNA was extracted from granulosa cells treated with melatonin in the absence/presence of preincubation with luzindole or 4P-PDOT for 48 h using RNAprep pure cell Kit (catalog number: DP430; Tiangen, Beijing, China) according to the manufacturer’s protocols. Moreover, RNase-free DNaseI was used to digest the RNA to remove the genomic DNA. The content of RNA was quantified by Nanodrop One (Thermo Scientific, Waltham, MA, USA). The cDNA was synthesized using a RevertAid First Strand cDNA Synthesis Kit (catalog number: K1622; Thermo Scientific, Waltham, MA, USA) according to the manufacturer’s protocols.

### Real-time PCR

The target genes expression level were carried out using the quantitative real-time PCR with LightCycler 480 SYBR Green I Master Mix (catalog number: 4887352001; Roche, Penzberg, Germany) according to our previously reported method (Wang et al., 2017b, 2018). The primer pairs designed for measuring the target genes were listed in Table 1. A total of 10 μL reaction volume was prepared as follows: 5 μL SYBR Green I Master Mix, 2 μL cDNA, 0.5 μM forward and reverse primers, and 2 μL RNase and DNase-free water. Real-Time PCR amplification was performed in a LightCycler 480 II System (Roche, Mannheim, Germany), and the procedure was as follows: 95 °C for 5 min, 40 cycles of 95 °C for 30 s, annealing at particular temperatures for 20 s, 72 °C for 20 s. To confirm specific PCR product, the melting curve from 65 °C to 95 °C was performed after real-time PCR reactions. Normalization was performed using β-actin as a control in each sample. The date analysis was using the 2^−∆∆Ct^ method (Livak & Schmittgen, 2001).

**Table 1:** Sequences of primer pairs for quantitative real-time PCR.

Gene		Forward primer sequence (5′→3′)	Reverse primer sequence (5′→3′)	Length
*Bax*	TGCAGAGGATGATCGCAGCTGTG		CCAATGTCCAGCCCATCATGGTC	198
*Bcl2*	CGCATCGTGGCCTTCTTTGAGTT		GCCGGTTCAGGTACTCAGTCAT	115
*Bcl-xl*	ATGGCAGCAGTAAAGCAAG		GCTGCATTGTTCCCATAGA	236
*Caspase-3*	*CAGACAGTGGTGCTGAGGATGA*		*GCTACCTTTCGGTTAACCCGA*	211
*p53*	CCTCCCAGAAGACCTACCCT		CTCCGTCATGTGCTCCAACT	221
*GPX4*	TGTGCTCGCTCCATGCACGA		CCTGGCTCCTGCCTCCCAA	224
*SOD1*	GCTGTACCAGTGCAGGTCCTCA		CATTTCCACCTCTGCCCAAGTC	228
*CyclinD1*	GCCCTCGGTGTCCTACTTCAA		ACAGGAAGCGGTCCAGGTAGT	152
*CyclinE1*	CCTCCAAAGTTGCACCAGTT		AGGATACTGAGGCAGGAGCA	195
*P21*	CGTCTCAGGAGGACCACTT		TCAGTCTGCGTTTGGAGTG	159
*β-actin*	CATCGGCAATGAGCGGTTCC		CCGTGTTGGCGTAGAGGTCC	145

### Western blot Analysis

Western blot analysis was performed as previously described else where (Wang et al., 2017b, 2018). Granulosa cells were collected after treatment for 48 h and lysed in RIPA buffer (catalog number: 89900; ThermoFisher, Rochford, IL, USA), then denatured by boiling for 5 min with bromophenol blue and frozen at −80 °C. The proteins were separated by 12% polyacrylamide gel electrophoresis, and then transferred to polyvinylidene fluoride membrane (Millipore, Bedford, MA, USA). Firstly, the membrane were incubated with primary antibody: Bcl2 mouse monoclonal antibody (1:500, sc-7382; Santa Cruz, Dallas, TX, USA), Bax mouse monoclonal antibody (1:500, sc-20067; Santa Cruz, Dallas, TX, USA), caspase-3 rabbit polyclonal antibody (ab13847; Abcam, California, USA) and β-actin mouse monoclonal antibody (1:1000, SC-47778; Santa Cruz, Dallas, TX, USA). Later, the membrane was detected by incubation with HRP labeled goat anti-rabbit secondary antibody (SC-2054; Santa Cruz, Dallas, TX, USA) or goat anti-mouse secondary antibody (1:5000; SC-2005; Santa Cruz, Dallas, TX, USA), respectively. Finally, membranes was incubated with the Clarity Western ECL kit (catalog number: 170-5060; Bio-Rad Laboratories, Hercules, CA, USA), and scanned in a ChemiDocXRS chemiluminescent imaging system (Bio-Rad, Hercules, CA, USA).

### Experimental design

To assess the potential effects of melatonin on granulosa cells, melatonin was added to medium at high dose concentrations (10^−5^ M) and low dose concentrations (10^−9^ M). The mRNA level of apoptosis related genes (*Bcl2*, *Bcl-xl*, *Bax*, *caspase-3* and *p53*), cell cycle associated gene (*CyclinD1*, *CyclinE1* and *P21*) as well as other genes (*SOD1* and *GPX4*) involved in antioxidation were detected by real-time quantitative PCR. To confirm whether melatonin inhibited granulosa cells apoptosis via its receptor, melatonin, melatonin plus 4P-PDOT or luzindole were added to medium in each experimental group, respectively. Therefore, experimental groups were divided as following: DMSO group; melatonin group; melatonin plus 4P-PDOT group; and melatonin plus luzindole group. Furthermore, in each experimental group, we detected the expression level of apoptosis related genes (*Bcl2*, *Bcl-xl*, *Bax*, *caspase-3* and *p53*), cell cycle associated gene (*CyclinD1*, *CyclinE1* and *P21*) and antioxidation related genes (*SOD1* and *GPX4*). In addition, we also detected protein expression level by western blot. Melatonin, 4P-PDOT, and luzindole were dissolved in DMSO, and then diluted to the final concentration (10^−5^ M and 10^−9^ M) with DMEM (catalog number: 430-2100; Gibco, Grand Island, NY, USA) before adding to the cultured granulosa cells. DMSO was added to medium as a control at the same concentration.

### Statistical analysis

All data were presented as Mean ± SEM of triplicate experiments (*n* = 3). Significant difference was evaluated using Duncan’s multiple comparisons following one-way ANOVA with the General Linear Models Procedure of Statistical Analysis Systems (SAS Inc., Cary, NC, USA). *P* < 0.05 was considered significant.

## Results

### Effects of melatonin and melatonin receptor antagonist supplementation on the cell cycle related genes

**Figure 1:**
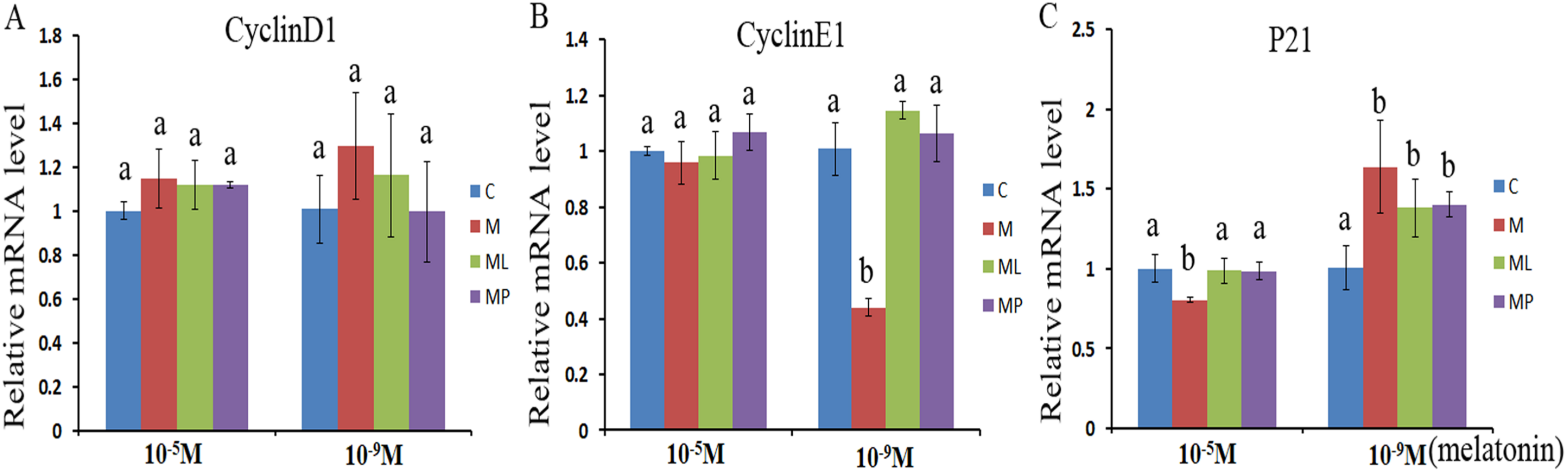
Effects of high dose (10^−5^ M) and low dose (10^−9^ M) melatonin and melatonin receptor antagonist supplementation on the cell cycle related genes (*CyclinD1*, *CyclinE1* and *p21*). The mRNA levels of *CyclinD1* (A), *CyclinE1* (B) and *P21* (C) were examined by real-time PCR in granulosa cells at 48 h after melatonin supplementation in the absence/presence of luzindole or 4P-PDOT. The quantity of mRNA was normalized to that of β*-actin*. The statistical differences were performed using one-way ANOVA. Statistical differences among the samples were labeled with different letters (*P* < 0.05). About 60 bovine ovaries were used in this study. The experiment was repeated three times independently. Abbreviations: C, control; M, melatonin; ML, melatonin and luzindole; MP, melatonin and 4P-PDOT.

The effects of melatonin within high dose (10^−5^ M) and low dose (10^−9^ M) on regulation of cell cycle processes were investigated by evaluating the expression of cell cycle related genes (*CyclinD1*, *CyclinE1* and *P21*) after melatonin supplementation in the absence/presence of luzindole or 4P-PDOT (Fig. 1). The expression of *CyclinD1*, *CyclinE1* and *P21* were not significantly altered after melatonin supplementation in high dose (10^−5^ M) with or without luzindole or 4P-PDOT compared with control groups (Figs. 1A–1C, *P* > 0.05), except *P21* in the melatonin group (Fig. 1C, *P* < 0.05). Moreover, *CyclinD1* and *CyclinE1* expression was not differ and *P21* was significantly different among melatonin group, melatonin plus luzindole group and melatonin plus 4P-PDOT group (Figs. 1A–1C, *P* > 0.05). The expression of *CyclinD1*, *CyclinE1* and *P21* in low dose (10^−9^ M), however, were different to that of high dose (10^−5^ M) (Figs. 1A–1C). *CyclinD1* and *CyclinE1* expression were not significantly different after melatonin supplementation with luzindole or 4P-PDOT compared with control groups (Figs. 1A and 1B, *P* > 0.05). The expression of *P21* was significantly enhanced after melatonin supplementation with or without luzindole or 4P-PDOT compared with control groups (Fig. 1C, *P* < 0.05), and there was no significant difference among melatonin group, melatonin plus luzindole group and melatonin plus 4P-PDOT group (Fig. 1C, *P* > 0.05). Moreover, the expression of *CyclinE1* was significantly inhibited after melatonin supplementation (Fig. 1B, *P* < 0.05). These results demonstrated that high dose (10^−5^ M) melatonin inhibited the *P21* expression, which were affected by melatonin receptor antagonist, luzindole and 4P-PDOT supplementation. However, low dose (10^−9^ M) melatonin increased the expression of *P21* to regulate the cell cycle, which were not affected by melatonin receptor antagonist, luzindole and 4P-PDOT supplementation.

### Effects of melatonin and melatonin receptor antagonist supplementation on granulosa cells apoptosisrelated genes

**Figure 2:**
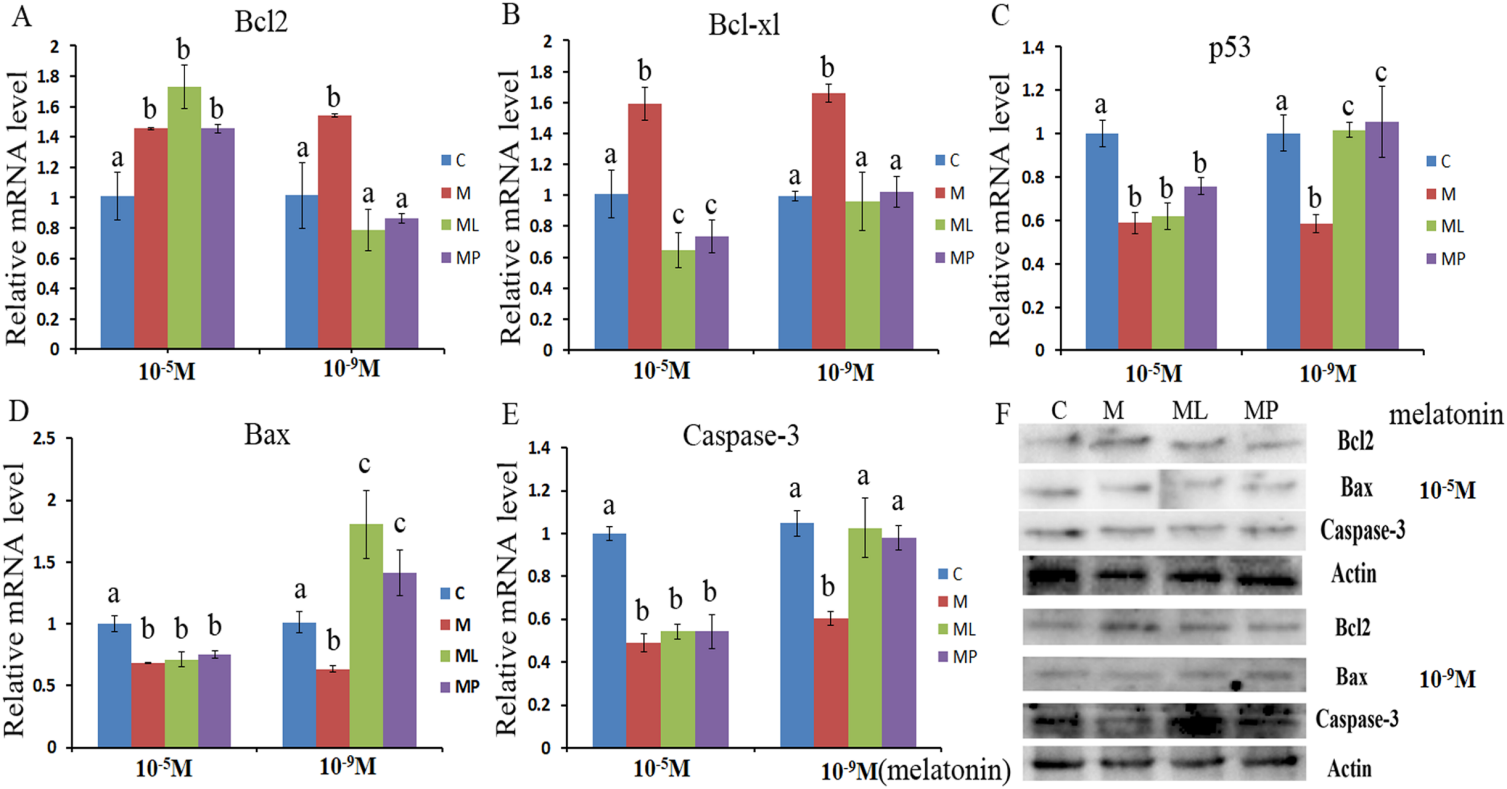
Effects of high dose (10^−5^ M) and low dose (10^−9^ M) melatonin and melatonin receptor antagonist supplementation on granulosa cells apoptosis related genes (*Bcl2*, *caspase-3*, *Bcl-xl*, *Bax* and *p53*). The mRNA abundance of *Bcl2* (A), *Bcl-xl* (B), *p53* (C), *Bax* (D) and *caspase-3* (E) were examined by real-time PCR at 48 h and corresponding protein abundance (F) were detected by Western blot after melatonin supplementation in the absence/presence of luzindole or 4P-PDOT. mRNA abundance was normalized to that of β*-actin*. The statistical differences were performed using one-way ANOVA. Statistical differences among the samples were labeled with different letters (*P* < 0.05). About 60 bovine ovaries were used in this study. The experiment was repeated three times independently. Abbreviations: C, control; M, melatonin; ML, melatonin and luzindole; MP, melatonin and 4P-PDOT.

The effects of melatonin in high dose (10^−5^ M) and low dose (10^−9^ M) on regulation of apoptosis related genes, including *Bcl2*, *caspase-3*, *Bcl-xl*, *Bax* and *p53*, were investigated (Fig. 2). The results showed that melatonin supplementation significantly decreased the expression level of *caspase-3*, *p53* and *Bax*, while increasing the expression level of *Bcl2* and *Bcl-xl* (Figs. 2A–2E, *P* < 0.05).These results revealed that melatonin inhibited granulosa cells apoptosis through regulating apoptosis related genes.

To further confirm whether melatonin inhibits granulosa cells apoptosis via its receptor, the expression level of *Bcl2*, *Bcl-xl*, *caspase-3*, *Bax* and *p53* were detected after granulosa cells treated with melatonin within 4P-PDOT or luzindole. The expression of *Bcl-xl*, *caspase-3*, *Bax* and *p53* were significantly downregulated and *Bcl2* expression level was significantly upregulated in the melatonin plus luzindole group and melatonin plus 4P-PDOT group with high dose (10^−5^ M) compared with control group (Figs. 2A–2E, *P* < 0.05). Further analysis found that there were no significant difference among melatonin group, melatonin plus luzindole group and melatonin plus 4P-PDOT group in the expression of *Bcl2*, *caspase-3*, *Bax* and *p53* (Figs. 2A, 2C–2E, *P* > 0.05) with high dose (10^−5^ M). In contrast, the expression of Bcl-xl was significantly decreased in the melatonin plus luzindole group and melatonin plus 4P-PDOT group compared with high dose (10^−5^ M) melatonin group (Fig. 2B, *P* < 0.05). These results indicated that the effect of high dose (10^−5^ M) melatonin on regulating the expression of *Bcl2*, *caspase-3*, *Bax* and *p53* was not affected by the melatonin receptor antagonist, luzindole and 4P-PDOT. As for low dose (10^−9^ M), melatonin plus luzindole group and melatonin plus 4P-PDOT group significantly promoted the expression level of *Bax* and inhibited the expression level of *Bcl2* compared wiht control group (Figs. 2A and 2D, *P* < 0.05). The expression level of *caspase-3*, *Bcl-xl* and *p53* were not significant difference in the melatonin plus luzindole group and melatonin plus 4P-PDOT group compared with control group (Figs. 2B, 2C and 2E, *P* > 0.05). Moreover, the expression level of *Bcl2*, *Bcl-xl*, *caspase-3*, *Bax* and *p53* were significant difference in melatonin group compared with that of melatonin plus luzindole group and melatonin plus 4P-PDOT group (Figs. 2A–2E, *P* < 0.05). Therefore, melatonin receptor antagonist, luzindole and 4P-PDOT indeed could affect the effect of low dose (10^−9^ M) melatonin on regulating apoptosis related genes.

### Effects of melatonin and melatonin receptor antagonist supplementation on granulosa cells antioxidant related genes expression

**Figure 3:**
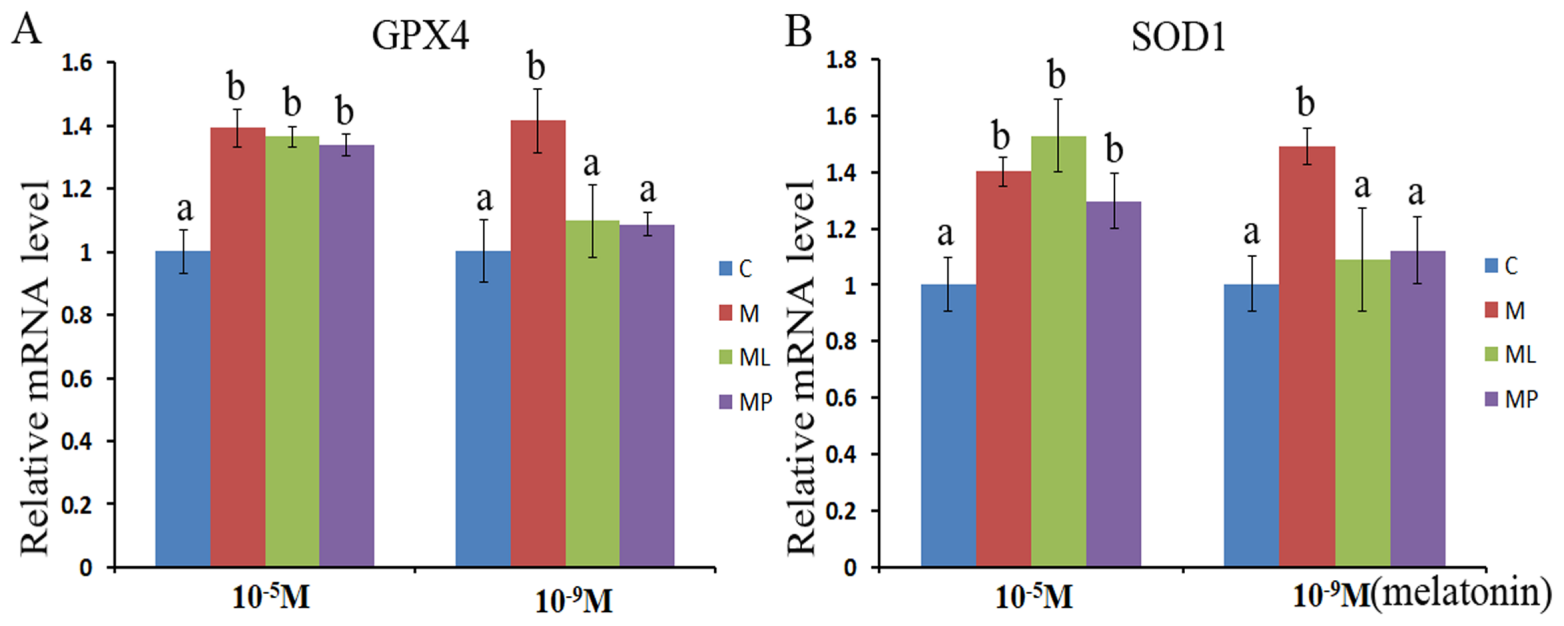
Effects of high dose (10^−5^ M) and low dose (10^−9^ M) melatonin and melatonin receptor antagonist supplementation on granulosa cells antioxidant related genes expression (*SOD1* and *GPX4*). The mRNA abundance of *GPX4* (A) and *SOD1* (B) were examined by real-time PCR at 48 h after melatonin supplementation in the absence/presence of luzindole or 4P-PDOT. mRNA abundance was normalized to that of β*-actin*. The statistical differences were performed using one-way ANOVA. Statistical differences among the samples were labeled with different letters (*P* < 0.05). About 60 bovine ovaries were used in this study. The experiment was repeated three times independently. Abbreviations: C, control; M, melatonin; ML, melatonin and luzindole; MP, melatonin and 4P-PDOT.

The *SOD1* and *GPX4* expression level was assessed after melatonin supplementation with or without luzindole or 4P-PDOT in bovine granulosa cells. The expression level of *GPX4* and *SOD1* both were significantly upregulated after melatonin supplementation in high dose (10^−5^ M) with or without luzindole or 4P-PDOT compared with control group (Figs. 3A and 3B, *P* < 0.05). Moreover, no change was observed between the melatonin plus luzindole and melatonin plus 4P-PDOT compared with the control group (Figs. 3A and 3B, *P* > 0.05). Furthermore, the expression of *SOD1* and *GPX4* were significantly promoted in the low dose (10^−9^ M) melatonin group compared with control group, melatonin plus luzindole group and melatonin plus 4P-PDOT group, and there was no significantly different among the melatonin plus luzindole, melatonin plus 4P-PDOT groups and control (Figs. 3A and 3B, *P* < 0.05 ). Therefore, melatonin inhibited oxidative stress in bovine granulosa cells through modulating the expression level of *SOD1* and *GPX4*, and melatonin receptor antagonist, luzindole and 4P-PDOT could affect low dose melatonin mediating the expression of *SOD1* and *GPX4*, however, did not alter the high dose melatonin effect on modulating the expression of *SOD1* and *GPX4* in bovine granulosa cells.

## Discussion

Ovarian follicle continuous development process give animals the chance to procreate, however, many factors affect the ovarian follicle development process, and cause most of the follicle undergo atresia. The high concentration melatonin is present in follicular fluid, and also it has been found that melatonin plays an important role in follicular development. In addition to melatonin, granulosa cells also is an important factor, which synthesize an array of factors, such as steroids, growth factors and cytokines, and affect the oocytes mature and follicle development. However, how melatonin affects the effects of bovine granulosa cells through its receptors is still needed to be investigated. Therefore, we tried to investigate the effect of melatonin with regard to bovine granulosa cells cell cycle, antioxidant and apoptosis, especially which receptor mediating the effects of melatonin on bovine granulosa cells. Moreover, we further revealed which receptor affected the effects of melatonin on granulosa cells by melatonin receptor antagonist supplementation.

*CyclinD1* and *CyclinE1* are involved in regulating of the cell cycle, and responsible for mediating the progression from phase G1 to S (M’baye et al., 2015; Zhen et al., 2014; Liu et al., 2018). On the contrary, *P21* is a cyclin-dependent kinase inhibitor and inhibits the progression from phase G1 to S (Wang et al., 2012; Liu et al., 2018; Han et al., 2013). In the present study, the expression of *CyclinD1* and *CyclinE1* were not significantly altered after melatonin supplementation in high dose (10^−5^ M), and just inhibited the expression of *P21*. However, the low dose (10^−9^ M) melatonin decreased the *CyclinE1* expression and increased the *P21* expression. Melatonin significantly promoted mesenchymal cells proliferation, and induced the cell arrest at G1 phase (Liu et al., 2011; Yang et al., 2017; Pan et al., 2018). Melatonin decreased the number of T lymphocytes cells in the G1/M phase in the patients with premature ovarian failure (Li et al., 2016) and promoted breast cancer cell apoptosis through blocking the G1/S transition (Cos et al., 1991). Therefore, melatonin exhibiting different effect may be due to the cell development state, such as, mouse oocyte vitrification arrested at the G1/S transition in parthenogenetic zygotes. However, melatonin significantly increased G1/S transitionin parthenogenetic zygotes for oocyte activation/embryonic development (Pan et al., 2018). During ovulation, granulosa cells were arrested at G0/G1 (Green et al., 2000), however, melatonin played a direct role in regulating progesterone production, LH receptor, GnRH and GnRH receptor gene expression in human granulosa-luteal cells (Woo et al., 2001). Moreover, the effect of melatonin on cell cycle was related to physiological state, such as health or pathology (Pan et al., 2018). Furthermore, melatonin receptor antagonist, luzindole and 4P-PDOT altered the effect of melatonin in high dose (10^−5^ M) and low dose (10^−9^ M) on the expression of *P21*, and affected the effect of melatonin on the expression of *CyclinE1*. It mean that the contradictory effects of melatonin receptor on mediating the melatonin on modulating the cell cycle may be related to the different melatonin dose and the mechanism is need to be further elucidated.

Mammalian granulosa cells act as a crucial role during follicle development and oocyte growth and maturation. Granulosa cells apoptosis or survival is considered as a signal for follicle development and oocyte growth, thus granulosa cells are essential for cell proliferation, follicular development, oocyte maturation, follicle ultimately ovulates or undergoes atresia (Matsuda et al., 2012; Choi et al., 2011; Tanabe et al., 2015; Kadariya et al., 2015; Chen et al., 2020). Even the granulosa cells state could determine the fate of follicle, mainly due to its effect on synthesize of steroids, growth factors, and cytokines, which have been proved to be essential for oocytes mature and follicle development (Matsuda et al., 2012; M’baye et al., 2015; Wang et al., 2017a, 2018; Chen et al., 2020). Therefore, improving the granulosa cells functions become very important for acting its physiological role during oocytes mature and follicle development. Recently, a large number of researches have focused on melatonin, which has exhibited its beneficial effects on anti-apoptosis and antioxidant. It was well known that the *Bcl-2* family play important role in mediating the cell apoptosis through modulating the expression of *Bcl2* and *Bax*. Melatonin was well established that its physiological functions in modulating the cell apoptosis was crucial for granulosa cells function, and thus acted as an important role in promoting follicular development and ovulates or inhibiting undergoes atresia (Matsuda et al., 2012; Choi et al., 2011; Kadariya et al., 2015). Moreover, melatonin had exhibited the anti-apoptotic effects in different cells by promoting the expression of *Bcl2* or inhibiting the expression of *Bax*, *p53* and *caspase3* and protecting the integrity of cells for maintaining optimal mitochondrial function (Fu et al., 2014; He et al., 2016b; Tan et al., 2013; Jou et al., 2010; Radogna et al., 2007, 2008, 2015). Our previous research also found that melatonin suppressed bovine granulosa cells apoptosis through mediating the expression of *Bcl2*, *Bcl-xl*, *Bax*, *p53* and *caspase3* (Wang et al., 2012, 2017b, 2018; Liu et al., 2018). Consistent with previous reports, in the present study, the high dose (10^−5^ M) and low dose (10^−9^ M) melatonin both could promote the expression of *Bcl2* and *Bcl-xl* and inhibit the expression of *Bax*, *p53* and *caspase3*. Furthermore, we further investigated the melatonin receptor, MT1 and MT2 mediating the actions of melatonin on preventing the apoptosis of granulosa cells by melatonin receptor antagonist, luzindole and 4P-PDOT. Luzindole and 4P-PDOT could suppress the effect of low dose (10^−9^ M) melatonin on promoting the expression of *Bcl2* and *Bcl-xl* and inhibiting the expression of *Bax*, *p53* and *caspase3*. However, Luzindole and 4P-PDOT did not affect the effect of high dose (10^−5^ M) melatonin on promoting the expression of *Bcl2* and *Bcl-xl* and inhibiting the expression of *Bax*, *p53* and *caspase3*. These findings indicated that the effect of low dose (10^−9^ M) melatonin on granulosa cells apoptosis was mediated by MT1 and MT2, and the high dose (10^−5^ M) melatonin affected the granulosa cells apoptosis by other pathway, besides MT1 and MT2. How melatonin receptors modulate the effect of melatonin on granulosa cells apoptosis was not very clear. He et al. (2016b) research showed that MT2 mediate the effect of melatonin on modulating porcine granulosa cells proliferation and apoptosis. However, there was evidence that melatonin modulate the cell apoptosis through MT1 and MT2 acting in a complementary way (Radogna et al., 2007; Espino et al., 2010, 2011). Furthermore, our recent research indicated that MT1 and MT2 might work in concert to modulate bovine granulosa cells function by regulating cellular progression and apoptosis (Wang et al., 2017b, 2018; Liu et al., 2018).

Reactive oxygen species is another factor that can induce cell apoptosis and thus affect the follicular development and oocyte maturation (Matsuda et al., 2012; He et al., 2016a; Chappel, 2013). Melatonin not only directly scavenges the ROS by crossing all cells members, but also can improve anti-oxidant enzymes expression (Tamura et al., 2009; Reiter et al., 2002, 2014), which is consistent with its effects on promoting the survival of granulosa cells, follicular development, and maintaining pregnancy (Matsuda et al., 2012; Choi et al., 2011; Arosh et al., 2004; Cruz et al., 2014; Liu et al., 2018 ). In the present study, the high dose (10^−5^ M) and low dose (10^−9^ M) melatonin both could stimulate the expression of *SOD1* and *GPX4*. Moreover, melatonin receptor antagonist, luzindole and 4P-PDOT could affect the effect of low dose melatonin on promoting the expression of *SOD1* and *GPX4* in bovine granulosa cells. However, did not alter the high dose melatonin effect. Which were consistent with previous reports that melatonin increased the expressions of *GPX4* and *SOD1* in in bovine embryo (Wang et al., 2014), porcine oocyte (Li et al., 2015), porcine granulosa cells (He et al., 2016a, 2016b) and the mouse granulosa cells (Tanabe et al., 2015). Our recent research revealed that melatonin could stimulate the expression of *GPX4* and *SOD1* in bovine granulosa cells (Wang et al., 2017a, 2017b; Liu et al., 2018), which was not only depending on MT1 and MT2.

The effect of MT1 and MT2 is well cross talk in vivo and vitro research, and there are not consistent evidence on the role of MT1 and MT2 mediating the response to melatonin. MT1 and MT2 are considered to modulating complex reproductive mechanisms (Dubocovich & Markowska, 2005), however, they exhibit different roles. It has been demonstrated that MT1 plays critical role in regulating mammal gonadal activity (Yasuo et al., 2009). The MT1 receptor is sufficient and necessary to mediate effects of photoperiod-driven changes in melatonin on seasonal changes in behavior and reproductive function in a reproductively photoperiodic mammal (Prendergast, 2010). As well as melatonin and MT1 signaling transfer human chorionic gonadotropin stimulating information to ovary and play important role in regulating female reproduction (He et al., 2016c). On the other hand, melatonin modulates the porcine granulose cells apoptosis and proliferation through its receptor MT2 (He et al., 2016a). In the knockout mice, MT2 modulates the behavioral effect, immune and inflammatory responses (Drazen et al., 2001; Larson et al., 2006). Moreover, melatonin activates MT2 and then increases serum estradiol level and decreases ovarian GnIHR (Gonadotropin-inhibitory hormone receptor), which promote hens egg-laying rates (Jia et al., 2016). However, recent research indicate that MT1 and MT2 mediate the effect of melatonin through acting in a complementary way. Melatonin modulates the cell life/death balance via interaction with the MT1 and MT2 in human spermatozoa (Espino et al., 2010), human leucocytes (Espino, Rodríguez & Pariente, 2013) and U937 cell (Radogna et al., 2008), bovine granulose cells (Wang et al., 2018, 2017b; Liu et al., 2018). Consistrent with presient study, we also found that Luzindole and 4P-PDOT could block the effect of physiological doses (10^−9^ M) melatonin on cell cycle, apoptosis and antioxidant of bovine granulosa cells. Which indicate that MT1 and MT2 could mediate the effect of melatonin through acting in a complementary way in the bovine granulosa cells. What interests us most in this study is that the action mode of high dose melatonin and low dose melatonin were different. The high dose melatonin and low dose melatonin acted a uniform effect on inhibiting the antiapoptosis and enhancing antioxidation of bovine granulosa cells. However, the high dose (10^−5^ M) melatonin still inhibited the apoptosis and enhanced antioxidative relative genes expression within or without Luzindole and 4P-PDOT. Therefore, Luzindole and 4P-PDOT can not block the effect of high dose melatonin on granulosa cells. Luzindole and 4P-PDOT were initially considered as competitive melatonin receptor antagonists, which could antagonize melatonin potentiation effect. The concentration of 100 nM and above Luzindole or 4P-PDOT, acting as an inverse agonists at MT1 and MT2, exist in a constitutively active form in recombinant MT1 and MT2 systems. The mechanism that melatonin receptors are constitutively active is that Luzindole and 4P-PDOT couple to G protein in the presence or absence of their ligand (Browning et al., 2000; Witt-Enderby & Dubocovich, 1996; Roka et al., 1999; Glaser, Masana & Dubocovich, 1998; Dubocovich et al., 2003). Consistrent with these studies, the high dose (10^−5^ M) melatonin receptors antagonists, Luzindole and 4P-PDOT, indeed exhibit an agonists effect of melatonin on antiapoptosis and antioxidation of bovine granulosa cells. Therefore, the present study indicates how different both Luzindole and 4P-PDOT affect the regulatoin effect of melatonin on cell function. Nonetheless, Luzindole and 4P-PDOT serve as an excellent melatonin receptor antagonists showing pharmacological efficacies at the MT1 and MT2. Therefore the difference must be taken when investigating functional studies mediated by Luzindole and 4P-PDOT that are not fully identical in the target tissue under study.

## Conclusion

In the present research, we investigated the effects of melatonin supplementation on the cell cycle, apoptosis and oxidative stress in the bovine granulosa cells. Moreover, we further revealed which receptor affected the effects of melatonin on granulosa cells by melatonin receptor antagonist supplementation. The results showed that the high dose (10^−5^ M) and low dose (10^−9^ M) melatonin both could act as an essential role in modulating granulosa cells apoptosis, cell cycle and antioxidant. Furthermore, the beneficial effect could be related to mediate the expression of *Bcl2*, *Bcl-xl*, *Bax*, *caspase-3*, *p53*, *P21*, *SOD1* and *GPX4* in granulosa cells. Our study also emphasized that the effect of low dose (10^−9^ M) melatonin on granulosa cells apoptosis was mediated by MT1 and MT2, and the high dose (10^−5^ M) melatonin affected the granulosa cells apoptosis by other pathway, besides MT1 and MT2. Moreover MT1 and MT2 may work in concert to modulate bovine granulosa cells function by regulating cellular progression and apoptosis. The present study benefits to understand that MT1 and MT2 mediate the effect of melatonin on modulating granulosa cells functions.

## Supplemental Information

10.7717/peerj.10627/supp-1 Supplemental Information 1 Effects of high dose(10^−5^ M) melatonin and melatonin receptor antagonist supplementation on related genes expression. Raw data for Ct value of 10 target genes and 1 reference gene, cell cycle, cell apoptosis and antioxidant related genes in four experimental group: control, melatonin group, melatonin plus 4P-PDOT group and melatonin plus luzindole group.

10.7717/peerj.10627/supp-2 Supplemental Information 2 Effects of low dose(10^−9^ M) melatonin and melatonin receptor antagonist supplementation on granulosa cells related genes expression. Raw data for Ct value of 10 target genes and 1 reference gene, cell cycle, cell apoptosis and antioxidant related genes in four experimental group: control, melatonin group, melatonin plus 4P-PDOT group and melatonin plus luzindole group.

10.7717/peerj.10627/supp-3 Supplemental Information 3 Bcl2, Bax, Caspase-3 and Actin in the control, melatonin group, melatonin plus 4P-PDOT group and melatonin plus luzindole group. Detected by the Western blot.

## Additional Information and Declarations

### Data Availability

The following information was supplied regarding data availability: Raw data are available in Supplemental Files.
