# Supplementary figures and images for: Luzindole and 4P-PDOT block the effect of melatonin on bovine granulosa cell apoptosis and cell cycle depending on its concentration
Source: PeerJ. 2021 Mar 8;9:e10627. doi: 10.7717/peerj.10627 (PMC7950190; doi:10.7717/peerj.10627)

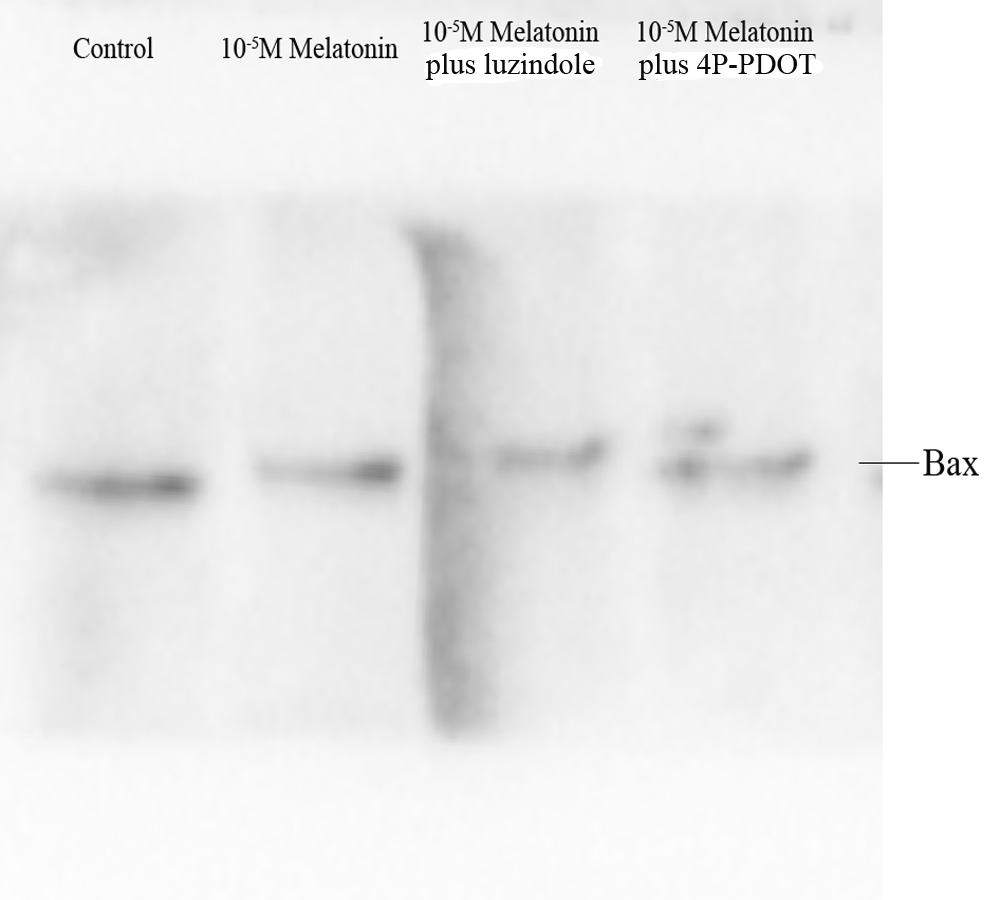

Supplement: Supplemental Information 3 [file peerj-09-10627-s003.zip › Supplemental Bax S2.png]

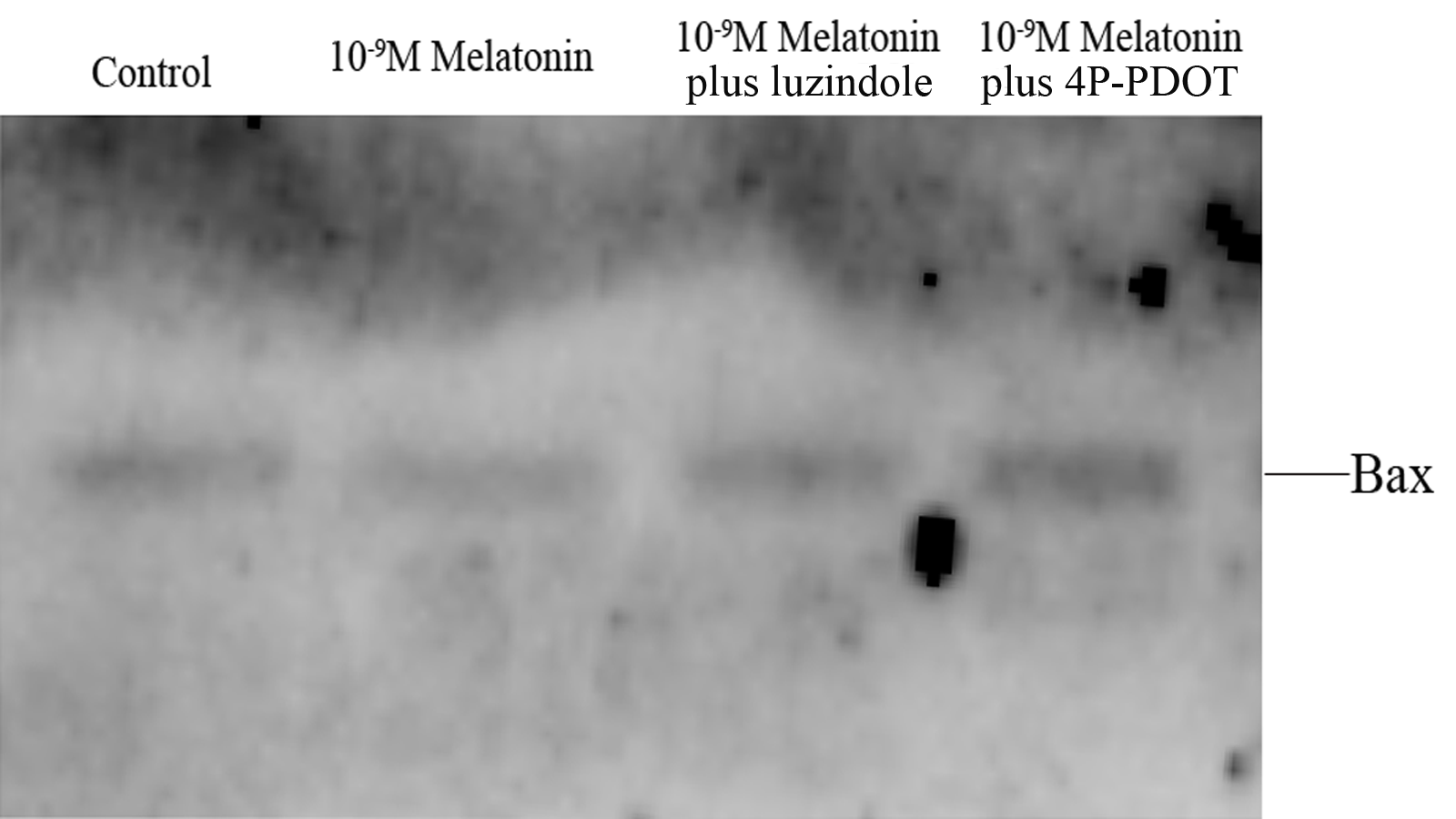

Supplement: Supplemental Information 3 [file peerj-09-10627-s003.zip › Supplemental Bax S6.png]

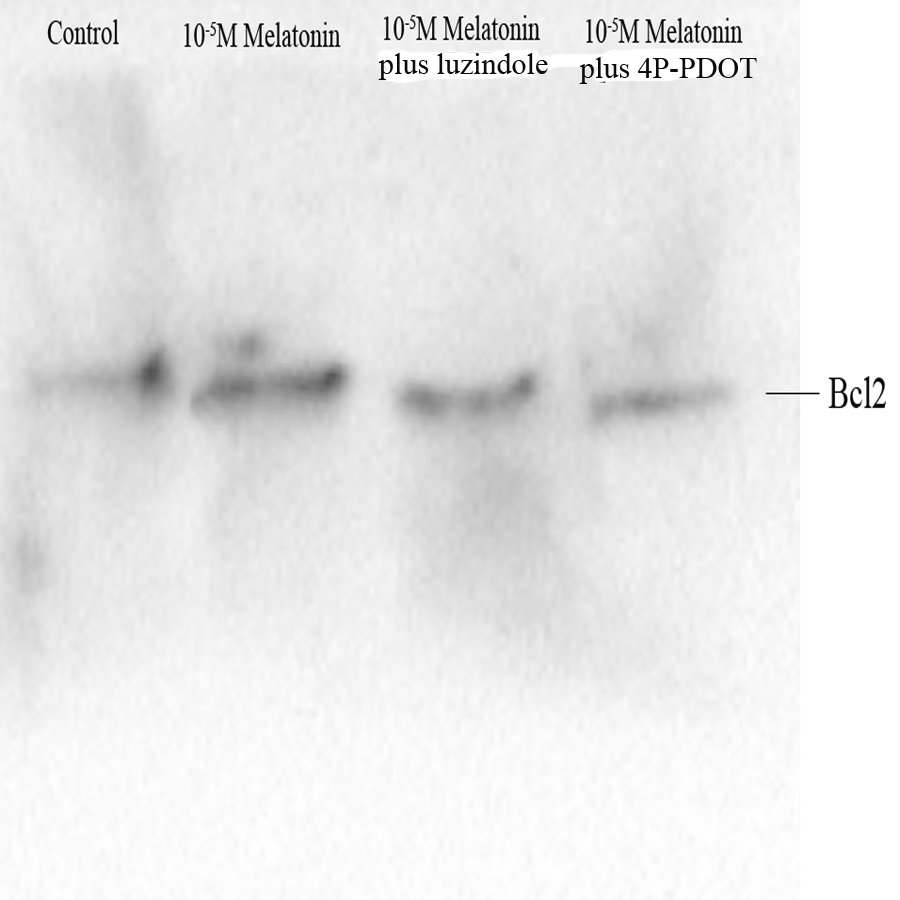

Supplement: Supplemental Information 3 [file peerj-09-10627-s003.zip › Supplemental Bcl2 S1.png]

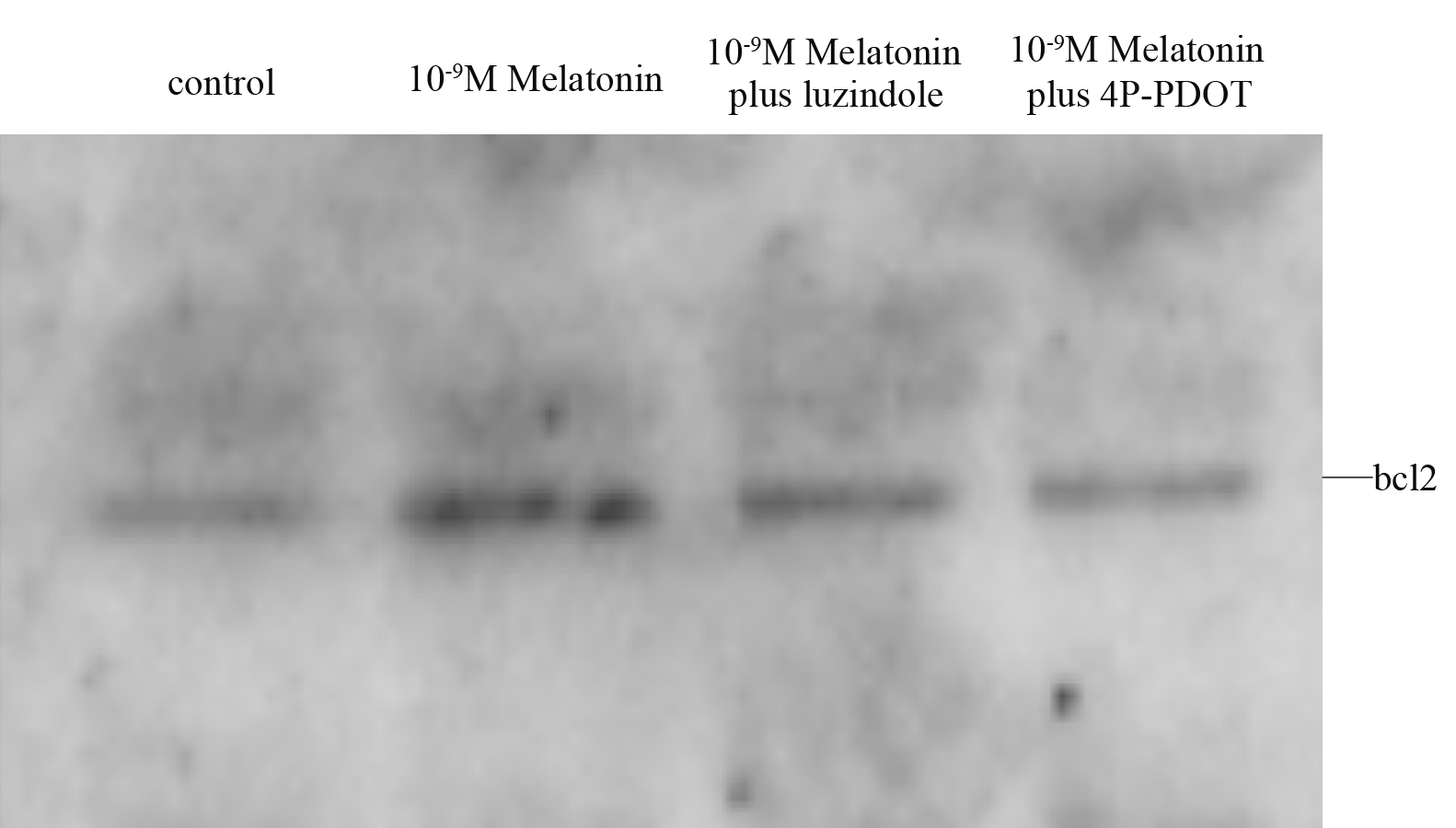

Supplement: Supplemental Information 3 [file peerj-09-10627-s003.zip › Supplemental bcl2 S5.png]

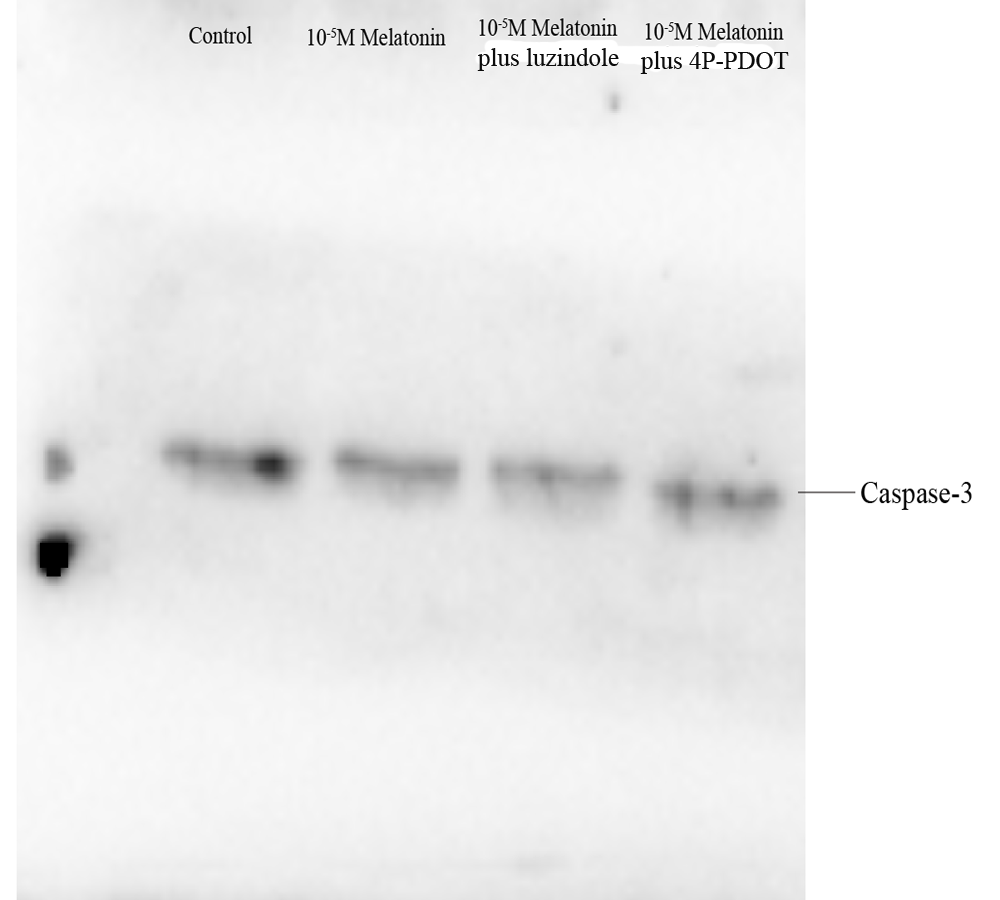

Supplement: Supplemental Information 3 [file peerj-09-10627-s003.zip › Supplemental caspase-3 S3.png]

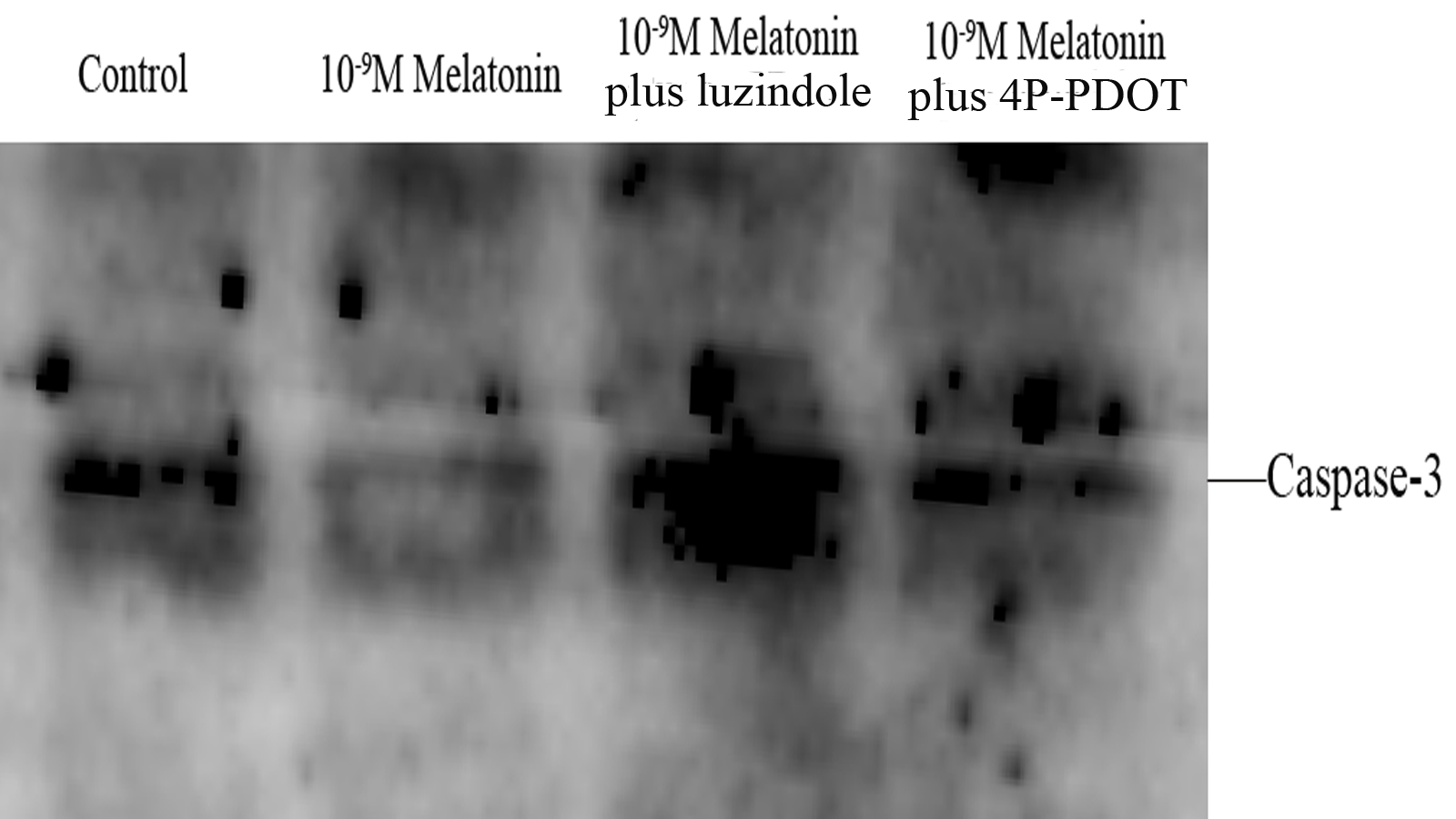

Supplement: Supplemental Information 3 [file peerj-09-10627-s003.zip › Supplemental caspase-3 S7.png]

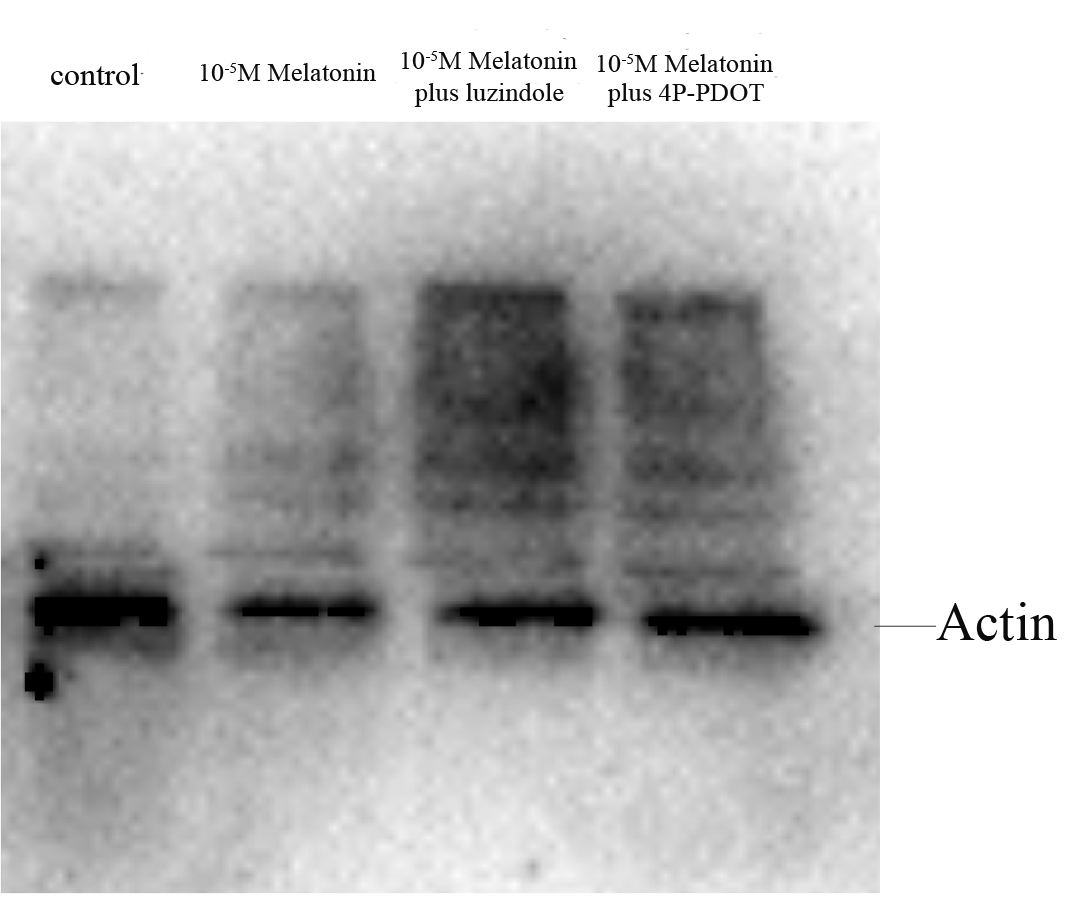

Supplement: Supplemental Information 3 [file peerj-09-10627-s003.zip › Supplemental actin S4.png]

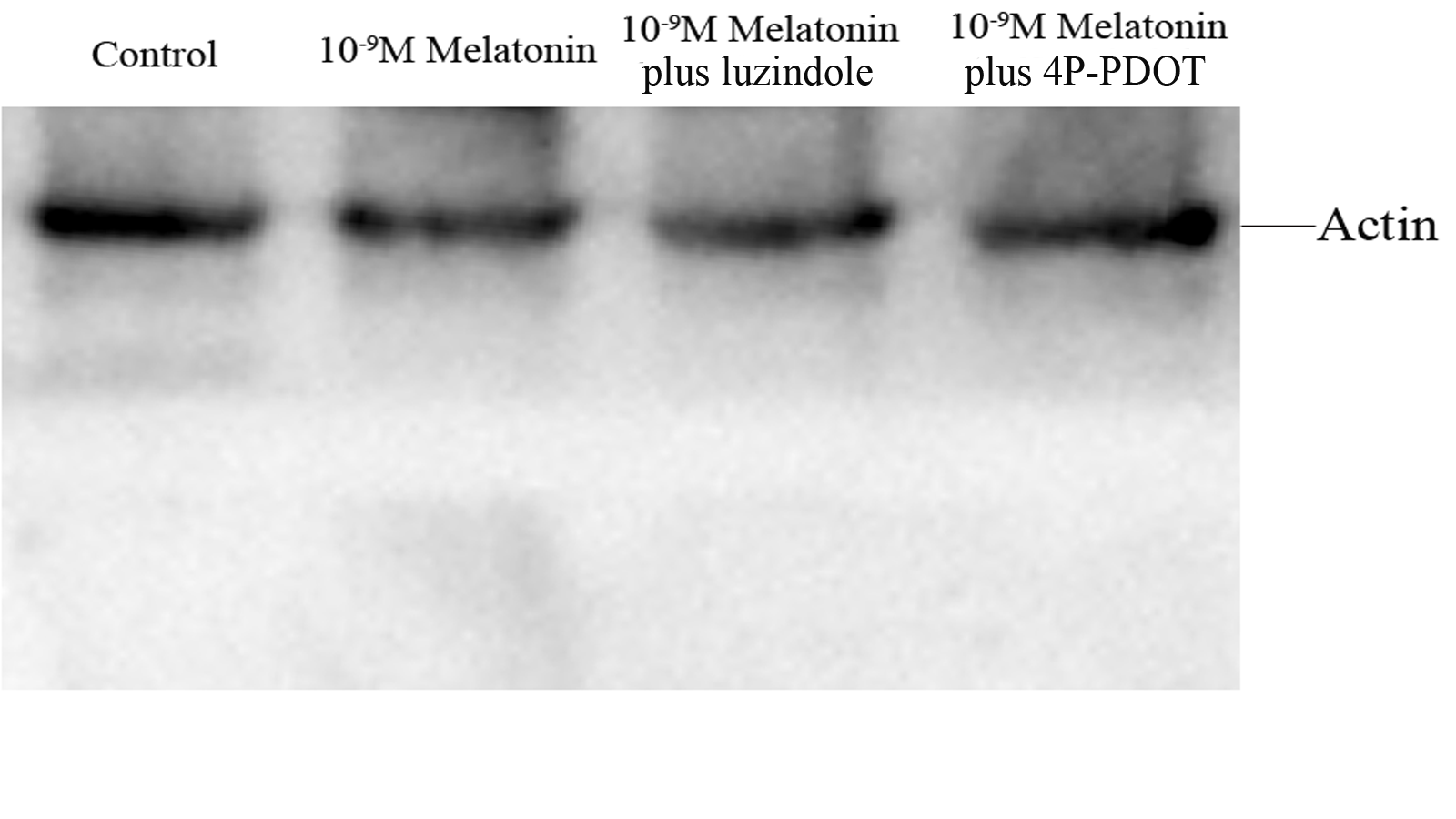

Supplement: Supplemental Information 3 [file peerj-09-10627-s003.zip › Supplemental ACTIN S8.png]
